# Investigation of the Interlaminar Shear Performance of Tufted Preforms and Composites under Mode II Loading Condition

**DOI:** 10.3390/polym14040690

**Published:** 2022-02-11

**Authors:** Chan Hui, Chen Chen, Xavier Legrand, Peng Wang

**Affiliations:** 1 University of Lille, Ensait, Gemtex, F-59000 Roubaix, France; chan.hui@ensait.fr (C.H.); xavier.legrand@ensait.fr (X.L.); 2 University of Haute-Alsace, Ensisa, Lpmt, F-68093 Mulhouse, France; peng.wang@uha.fr

**Keywords:** textile preform, composites, tufting, interlaminar shear, mode II loading

## Abstract

The influences of reinforcement by tufting on the interlaminar shear performance of laminated preforms and composites are studied in the present paper. A modified T steel shearing test was established and used to achieve a pure Mode II loading (sliding). Dry tufted preform (DTP) and cured tufted composites (CTC) with varied tufting spacing are considered for the understanding of the role of infused resin and the tufting density on the mechanical properties. Meanwhile, knowledge about the role of infused resins is gained. Additionally, cured tufted composites without threads (CT’C) were prepared under the identical tufting density to evaluate the effect of tufting threads. The results show that the denser the tufting density, the stronger the interlaminar shear strength of CTC, its improvement reaches 12% compared to the non tufted composites. However, the decreased effect also exists for the tufting spacing of 9 mm. Therefore, the tufting density needs to be optimized during the tufting process to improve the interlaminar shear properties of tufted reinforcement and composites. On the contrary, tufting without thread does not affect its mechanical properties compared to the non tufted composites.

## 1. Introduction

**Figure 1:**
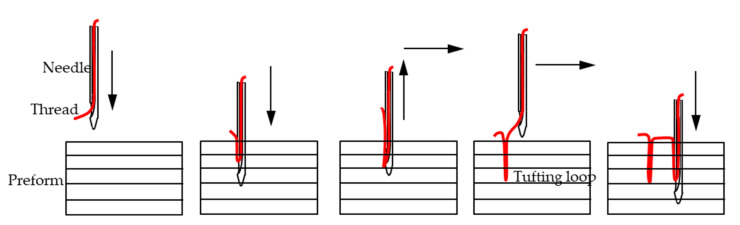
Aerotiss^®^ 03 tufting principle.

Advanced laminated textile reinforced composites have been extensively used in many industrial fields on account of their lightweight properties and better mechanical performance such as the higher in plane strength, stiffness, and resistance to fatigue compared to metallic materials. However, the conventional two-dimensional (2D) laminated structure shows unsatisfied interlaminar shear under mode I opening loading or mode II sliding loading because of the lack of linkage fibre positioned across the thickness direction [1,2,3]. A large number of studies conducted over the last two decades demonstrate that Through the Thickness Reinforcement (TTR) can meaningfully improve the resistance to delamination by inserting threads in the thickness direction [4,5,6,7,8]. TTR three-dimensional (3D) structure can be achieved through various approaches, including integrating 3D technologies such as 3D weaving, braiding, and knitting; and local 3D technologies such as z pinning, stitching, and tufting [7,9,10,11,12,13,14,15]. The enhancement of out of plane performance exists at the expense of in plane performance degradation. However, it also depends on the parameters of the TTR 3D reinforcement. Tufting is one of the significant local TTR technologies to assemble dry textile reinforcements or strengthen composites [16,17], which was first used in carpet fabrication and developed from stitching technology. In particular, tufting has been gradually applied in the fabrication of thicker and complex composites on account of its simple and efficient process [18,19]. Figure 1 shows that only one threaded needle penetrates the preform under low tension, and the thread is retained within the preform by simple friction when the needle retracts, while forming a tufting loop. Compared to the stitching process, one-thread access to only one side of the preform is required [13,20,21], thus the stitching effect on the in plane properties can be effectively reduced during the tufting process [22,23].

The present paper focuses on the factors which affect the mechanical performance of tufted laminated materials. The majority of researchers have reconnoitered and demonstrated the advantages of the tufted materials on mechanical performance, especially the delamination resistance. For instance, Bortoluzzi et al. [8] dedicated about 27% increased resistance to delamination of tufted composites compared to non tufted ones. Martins et al. [23] studied the impact as well as the compression after impact (CAI) behaviors of tufted composites. They mention that the delamination area of the tufted composites is decreased with the tufting density increasing compared to the non tufted composites. Both have been conducted under the specific delamination mechanism in mode II condition, which is reflected by sliding loading.

**Figure 2:**
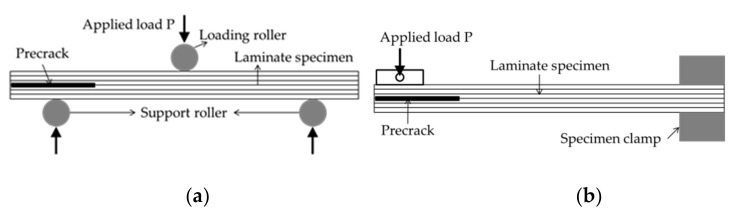
Schematics of (**a**) ENF test set-up and (**b**) ELS test set-up for mode II delamination resistance testing.

As per the literature, varying set ups can realize mode II delamination experimentation to present different mechanical performances. However, none of them has been standardized. In addition to the end notch flexure (ENF, see in Figure 2a) [24] and short beam bending which are used in the abovementioned delamination studies, the end loaded split (ELS, see in Figure 2b) [25] is also generally used. A modified T steel shearing test is proposed in the present study, which can explore the interlaminar shearing behavior of both tufted preforms and composites driven by the mode II sliding loading.

Nevertheless, there is currently a lack of research on tufted dry preforms. Moreover, since the TTR reinforcement of tufted composite sample consists of both thread and the resin surrounding it, the existing research does not analyze them respectively. This paper aims to assess under the Mode II loading, (i) the effect of tufting density on the interlaminar shear behavior of tufted preforms and tufted composites, respectively; (ii) the effect of tufting threads on the mechanical properties of tufted composites; and (iii) the role of the infusion resin in interlaminar shear performance. This investigation is conducive to optimize the mechanical performance of laminate composite and thus expand its application range in the industrial fields, especially in civil engineering, as the critical stressed structural components. To ensure the feasibility of the present study, samples with various tufting densities of dry tufted preforms (DTP), cured tufted composites with threads (CTC) and without threads (CT’C) were prepared in sequence. Meanwhile, the present modified T-steel interlaminar shear set up is introduced in detail.

## 2. Materials and Methods

### 2.1. Raw Materials

**Figure 3:**
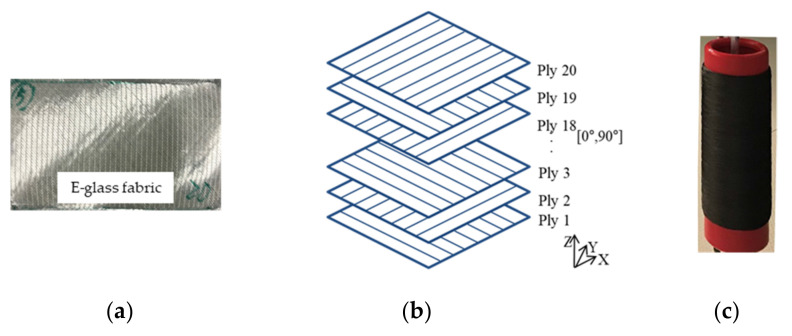
(**a**) E glass fabric; (**b**) schematic of the laminated preform; and (**c**) bobbin of carbon tufting threads.

Tufted preform or composite materials with and without Z reinforcement were fabricated using E glass Non-Crimp Fabric (NCF) with an areal density of 454.5 ± 5 g/m^2^ (see Figure 3a). Twenty layers of this NCF were then laid up in a stacking sequence with cross plies [0°/90°] (as shown in Figure 3b). The layers were assembled by a twisted carbon fiber thread as a tufting thread to enhance the interlaminar resistance. This thread is shown in Figure 3c and its parameters are given in Table 1.

### 2.2. The Preparation of Test Samples

**Figure 4:**
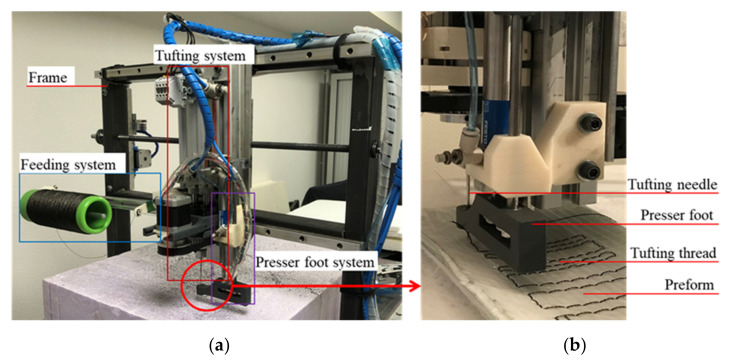
(**a**) Home-designed tufting device; (**b**) tufting process.

The DTP were carried out through a two step tufting process [26] according to the pre-set tufting parameters and configurations. Meanwhile, a classical tufting process was required to produce dry tufted preforms without thread, repeating the same tufting configuration as the DTP. In this process, a hollow needle without thread was applied to accomplish the tufting penetration by using a home designed tufting device (see Figure 4).

**Figure 5:**
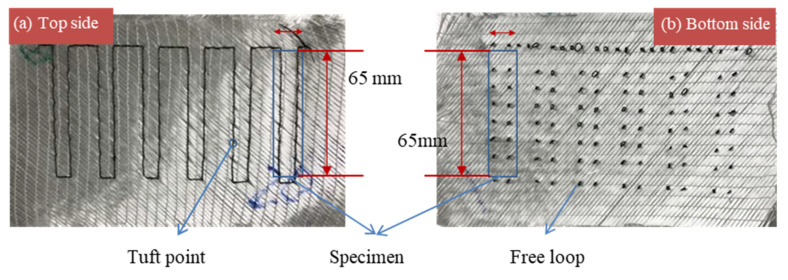
Dry tufted preform (**a**) top view and (**b**) bottom view using the tufting spacing of 9 mm.

Figure 5 shows the top and bottom sides of one of the DTP samples. Some of the samples obtained at this stage were be used to manufacture the next composite material (CTC and CT’C). In addition, the others were directly used for follow-up shearing testing after machining to size.

**Figure 6:**
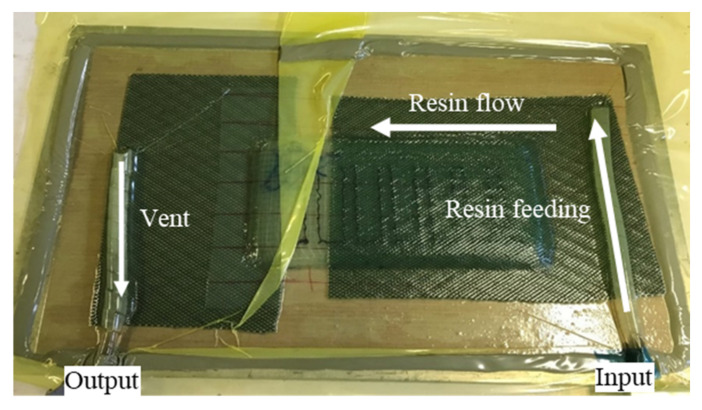
The tufted preform sample under the LRI process.

**Figure 7:**
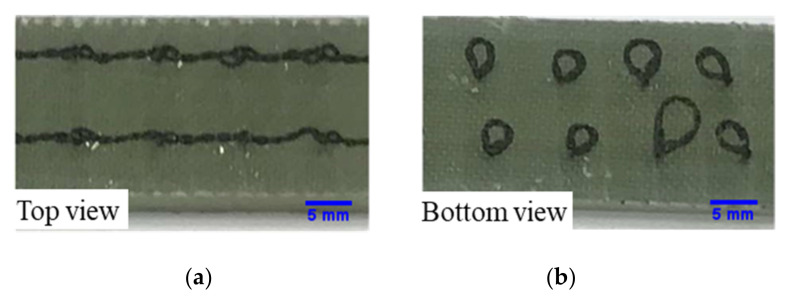
Cured tufted composite (**a**) top view and (**b**) bottom view.

Once the tufting process was completed, the TPwT preforms were vacuum infused with epoxy resin by using LRI (Liquid Resin Infusion) process (shown in Figure 6). The infused tufted composite was cured at room temperature for 48 h. Figure 7 demonstrates the top and bottom sides of an example of CTC samples.

To achieve the interlaminar shear test in the next section, it was necessary to machine the samples to the desired length of 65 mm and width 20 mm. The dry preform samples and the cured composite samples were respectively cut by electronic scissors and by water jet cutter. The variation in the thickness of the final samples was not large and can be ignored, and the value of their thickness was around 8 ± 0.5 mm. To understand the respective contribution of tufting thread, tufting density, the tufting action (typically only the tufting needle’s effect, not the thread’s), and cured resin on the interlaminar shearing performance, three different comparisons are proposed: (i) comparing, respectively, the DTP samples and CTC samples with different tufting spacing to study the effect of tufting density (tufting density is expressed by tufting spacing in the present paper); (ii) comparing CT’C samples and non-tufted composite samples to investigate the influence of pure tufting action; (iii) comparing CT’C and CTC samples to determine the role of cured tufting thread; (iv) comparing CTC samples and DTP samples to understand the contribution of cured resin. Initially, the non tufted was designed as a reference for both dry preforms and cured composites. However, for non tufted preform, as a reference in the DTP series, the lack of reinforcement in the Z direction (through the thickness), automatically resulted in delamination before the shear load was applied. There was no test result from this preform. Meanwhile, three various tufting spacing were evaluated: tufting spacing of 9, 6, and 3 mm corresponding to the 9 × 9, 6 × 6, and 3 × 3 mm^2^ square patterns, respectively. They are noted as T9, T6, and T3. The smaller the tufting spacing, the larger the tufting density, with T3 being the densest. Table 2 summarizes the parameters and manufacturing methods of all test samples, except the non tufted preform.

### 2.3. Interlaminar Shear Test Set-Up and Test Evaluation

**Figure 8:**
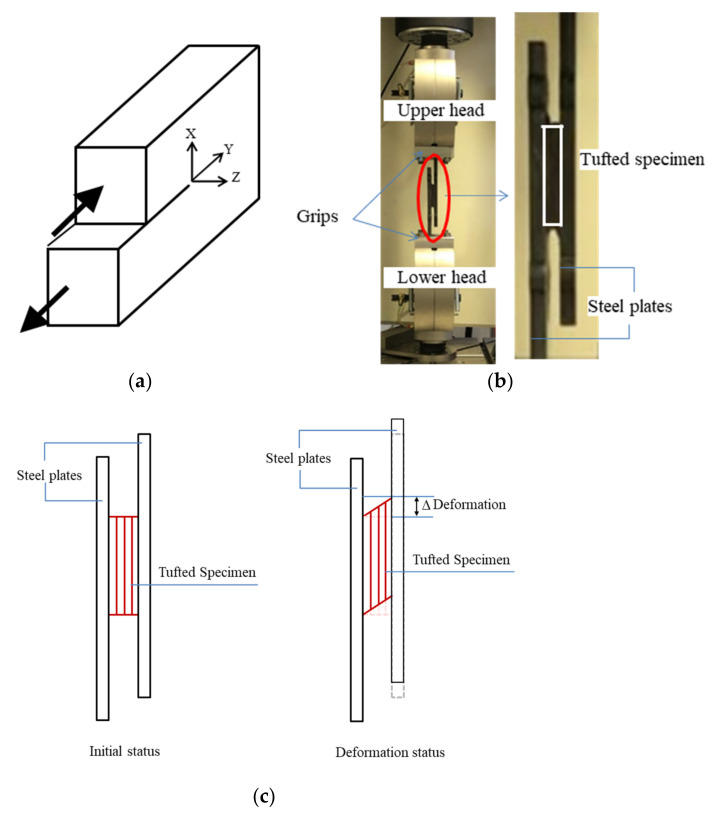
Interlaminar shear (**a**) mode II shear; (**b**) test set up; and (**c**) schematic view.

An interlaminar shear test fixture was required in the present chapter, and then the mode II in plane shearing (shown in Figure 8a) was chosen to achieve the interlaminar shear sliding behavior of the tufted samples. A T steel shear test fixture was designed as shown in Figure 8b and was optimized to be employed on the test samples with an aspect ratio of about 8 (length divided by thickness). A total of three series of 50 samples with different tufting spacing were tested applying this fixture to undergo the interlaminar shear investigation.

Concerning the interlaminar shear fixture in the present chapter, it was necessary to bond the sample onto two steel plates. Moreover, the bonding strength has to be strong enough between the sample and two plates to ensure that the effective shear failure occurred on the sample before the adhesive failed. Firstly, the steel plates needed to be cleaned with acetone just before the application of the adhesive. EC 9323B/A from 3M™ Scotch Weld™, an epoxy-based structural adhesive, was used to bond the tufting samples to the steel plates. The adhesive was cured in an oven at 65 °C for two hours and could offer shear strength up to 40 MPa at room test temperature according to its official technical data sheet, which was much higher than the ultimate shear strength of the samples after the preliminary test.

Interlaminar shear testing was performed in an INSTRON universal testing machine (type of 5985, Instron, Norwood, MA, USA, seen in Figure 8b). The test samples were performed under loading at a constant crosshead speed of 5 mm/min until failure. The representative data of load and displacement were traced during the whole testing process and the shear load–shear angle curve was plotted finally.

Following the present T steel shear fixture and the theoretical formula of shear behavior, the shear strength, τ, and the shear strain (here expressed by shear angle), ϵ, were calculated using the following formulas as Equations (1) and (2):

(1) $$\tau =\frac{P}{L\times W}$$

(2) $$\epsilon =\tan^{-1}\left(\frac{\Delta }{T}\right)$$

where, L, W, and T are the sample length, width, and thickness in mm, respectively; ∆ is the displacement of the steel plates (to express the specimen’s deformation which occurs during the shear testing, see Figure 8c) in mm; ϵ is the shear strain expressed by the shear angle measured in radians (rad), which is a non unit (shear strain is dimensionless); P is the shear load in N, when P reaches the peak point, then τ expresses the ultimate shear strength (in MPa).

## 3. Results

Following at least six repeated tests for each sample, all failures were caused by shearing delamination under mode II loading, the corresponding representative shear load–shear angle curves were then calculated on average. The results are given by three series samples: DTP, CT’C, and CTC, under the role of the respective tufting thread, tufting action, and cured tufting thread in the interlaminar shear test, in the following sections, respectively.

### 3.1. Interlaminar Shear Test of Dry Tufted Preform Samples

**Figure 9:**
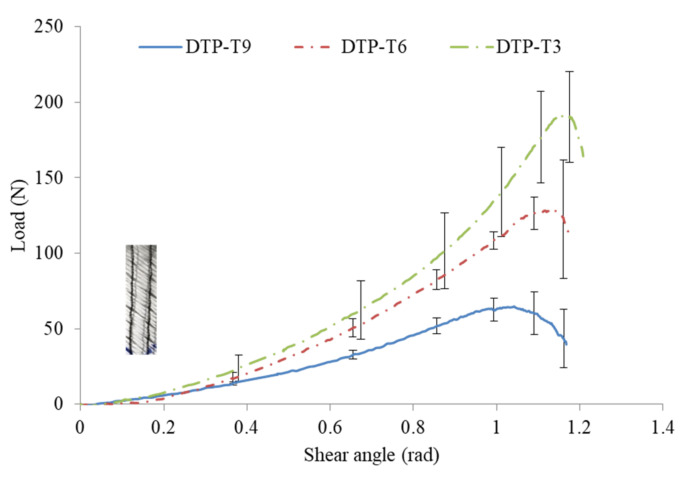
Representative shear load–shear angle curves of DTP specimens.

Figure 9 shows the representative shear load vs shear angle curves for the DTP samples with three tufting spacing of 9, 6, and 3 mm. It can be observed that the slopes of the three curves are slightly different. In particular, this difference becomes gradually increased after the shear angle of 0.4 rad until the failure. It is therefore considered that the stiffness of the DTP samples can be influenced by the tufting density. The results reveal the inversely proportional relationship between them. The smaller the tufting spacing, the denser the tufting density, and the stiffer the DTP samples. Regarding the maximum shear load, it can be observed that the average maximum shear loads are 65 ± 14.1 N, 129 ± 25.7 N, and 192 ± 30.1 N for DTP T9, DTP T6, and DTP T3, respectively.

**Figure 10:**
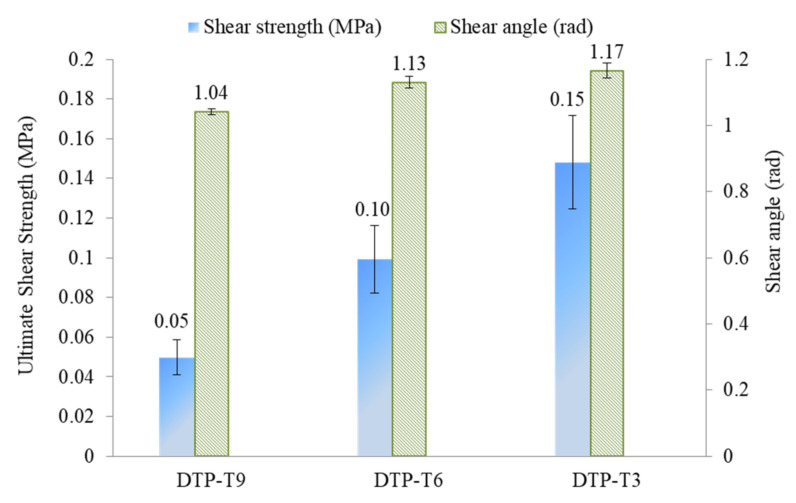
Representative shear properties of DTP specimens.

Furthermore, Figure 10 recaps the ultimate shear strength and the corresponding shear angle of DTP T9, DTP T6, and DTP T3, respectively. Regarding the ultimate interlaminar shear strength, the values are 0.05, 0.1, and 0.15 MPa for the DTP T9, DTP T6, and DTP T3, respectively. It increases with a tolerance of 0.05 MPa when the tufting spacing decreases with a tolerance of 3 mm. Regarding the shear angle at failure, it can be found that they are similar, with 1.04 ± 0.01, 1.13 ± 0.02, and 1.17 ± 0.02 rad for DTP T9, DTP T6, and DTP T3, respectively. It can be noted that tufting spacing has a remarkable effect on the interlaminar shear properties of DTP specimens. With the tufting spacing decreasing, the more intertwining between layers, the stronger the required shear load is.

### 3.2. Interlaminar Shear Test of Cured Tufted Composite Samples

Two series of cured tufted composites samples, CT’C and CTC, were investigated in the present section. Non-tufted composites were also prepared to be tested as a reference. Theire average shear failure load was 23,669 ± 232 N, the corresponding shear failure angle was 0.28 ± 0.01 rad, and 18.2 MPa of its ultimate shear strength. Then the results of CT’C and CTC are presented below.

#### 3.2.1. Cured Tufted Composite without Thread (CT’C)

**Figure 11:**
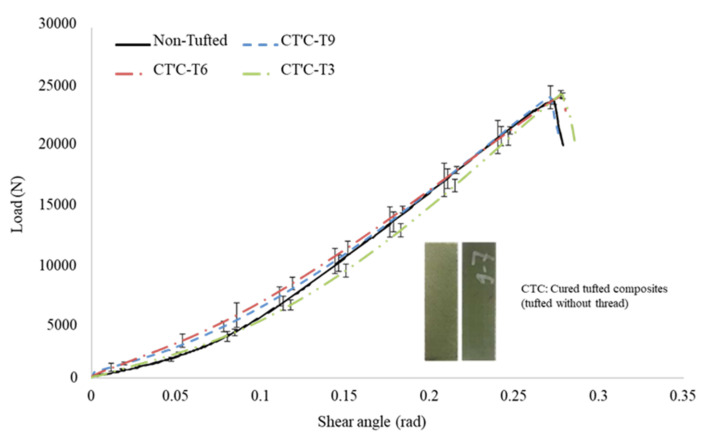
Representative shear load–shear angle curves of CT’C samples.

Figure 11 represents the results of the CT’C samples with three different tufting spacings and the reference non tufted samples. It is clear that the effect of tufting spacing on the shear load is too small. The maximum shear loads of T9, T6, and T3 are 24,044 ± 998, 24,170 ± 251, and 24,315 ± 305 N, respectively. Compared to the non tufted composites, the maximum load increases 1.6%, 2.1%, and 2.7%, respectively. The shear angles corresponding to the maximum load of all CT’C samples are similar to those of the non tufted composites, which are detected at about 0.28 ± 0.01 rad. Further results will be discussed in a later section.

#### 3.2.2. Cured Tufted Composite with Thread (CTC)

**Figure 12:**
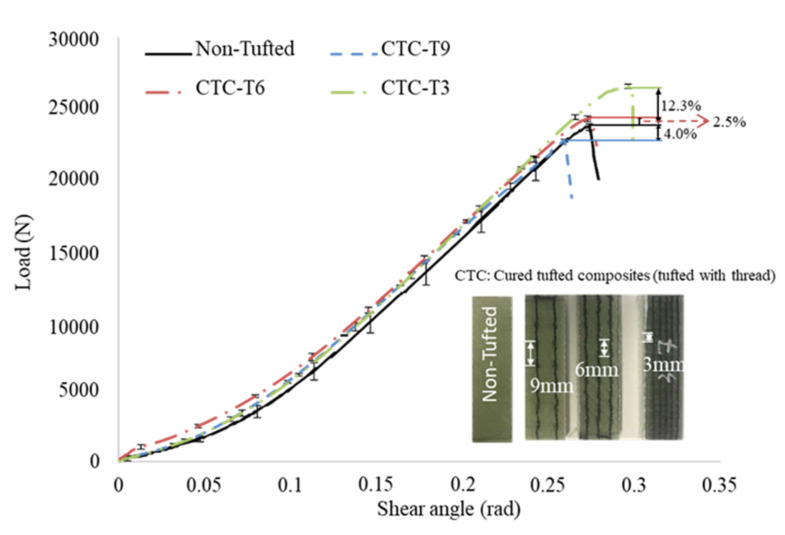
Representative shear load–shear angle curves of CTC samples.

As illustrated in Figure 12, the representative shear load vs shear angle curves of the CTC samples and the non tufted samples were calculated. This reveals that a higher interlaminar shear load is needed to delaminate the CTC samples. The average maximum shear loads are 22,723 ± 94, 24,256 ± 205, and 26,568 ± 152 N for T9, T6, and T3, respectively. Compared to that of the non tufted composite, the maximum shear load increases 2.5% and 12.3% for CTC T6 and CTC T3, respectively. Unfortunately, the value of CTC T9 decreases by 4.0%. It is also clear that the smaller the tufting spacing, the greater the shear angle needed to be reached at failure. The values are 0.26 ± 0.01, 0.27 ± 0.01, and 0.3 ± 0.01 rad for T9, T6, and T3, respectively. The shear angle changes −7.0%, −3.5%, and 7.0% compared to the non tufted composite samples. No doubt compared with the reference non tufted composites, the tufting presents both advantages and disadvantages of the maximum shear load and shear angle. Wherein, T6 is a critical tufting spacing to optimize the tufting parameters.

#### 3.2.3. Comparison Interlaminar Shear Strength

**Figure 13:**
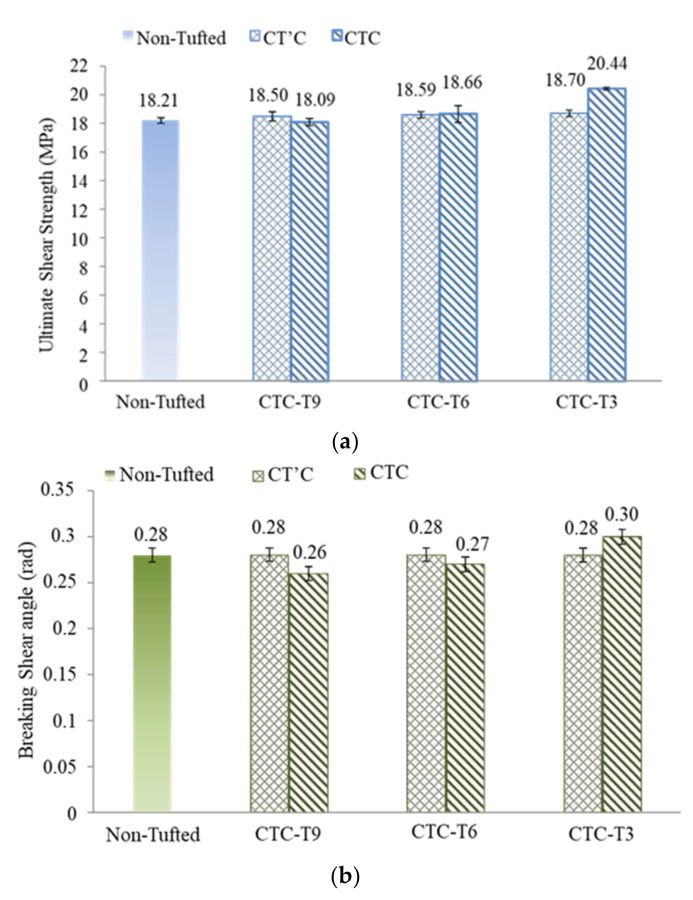
Comparison of (**a**) the maximum shear strength and (**b**) the shear angle at the failure between CT’C and CTC samples.

The comparison of interlaminar shear properties between CT’C and CTC samples are plotted in Figure 13. The comparison summarizes the ultimate interlaminar shear strength and the corresponding average shear failure angle. For the same tufting spacing, it can be noted that the improvement of the ultimate shear strength of CTC compared to the CT’C, with 0.4% and 9.3% improvement for tufting spacing 6 mm and 3 mm; however, 2.2% decrease for tufting spacing 9 mm. The corresponding shear angle increases by 7% and decreases by 7% respectively for the tufting spacing of 3 and 9 mm, 3.5% decreases for the tufting spacing of 6 mm.

## 4. Discussion

Table 3 summarizes the ultimate shear strength and the corresponding shear angle of all three series samples. It is generally proved that the presence of tufting thread can effectively improve the interlaminar shear performance of the laminated composite under mode II loading. The too-small differences in the interlaminar shear strength between the CT’C samples and the non tufted composites are negligible. The small differences in the interlaminar shear strength between the CT’C samples and the non tufted composites are negligible. Moreover, the CT’C samples with three different tufting densities yield the same average shear angle, equal to that of the non tufted composites, about 0.28 rad. It is believed that the pure tufting action (tufting without thread) does not significantly affect the interlaminar shear properties. This is due to the lack of threads in the thickness direction to offer the interlaminar reinforcement in this direction. This can rule out the positive effect of pure tufting action on the shear property when comparing the CTC samples and non-tufted composite samples. It also validates the improvement of shear strength being principally brought by the tufting thread. Therefore, the rationality and feasibility of the two step tufting method used in the present work are once again confirmed, i.e., a suitable guide needle without thread is placed before the tufting needle. However, it is considered that different tufting needles without threads (pure tufting action) may cause varying degrees of destruction to the preform structure when passing interlayers. It may bring a negative but also a positive effect, even no effect at all. It can nevertheless be controlled by combining the diameter of the tufting needle with the structure of the preform.

It is notable that only the CTC sample with tufting spacing of 9 mm shows lower ultimate shear strength than non tufted samples. In general, the presence of tufting thread effectively binds the laminated preform together to reduce the delamination and to increase the interlaminar shear strength of the tufted composites. However, i) as the penetration of the tufting thread, the laminate fibers are pushed apart, bringing a void which will be filled with resin during the LRI process to generate a resin rich zone [17,19,27], which may weaken the shear load-bearing capacity of CTC T9, causing earlier failure under mode ii sliding loading. ii) From the results of DTP, it is found that the shear strength of T9 is about one-third of T3, much weaker than the others, the destruction to the layer-to-layer or/and inter layers of the laminate preform may occur with the tufting thread passing through the layers. The quantity of tufting thread of T9 may not be sufficient to avoid delamination. Therefore, CTC T9 may fail due to the weakening of mode II sliding load-bearing, before the tufting thread plays its interlaminar enhancement. Tufting density is a remarkable factor to optimize the tufting parameters of the final composites. T6 is a critical tufting density to distinguish the positive and negative effects on the present interlaminar shear properties.

By comparing the results between DTP specimens and CTC specimens, it is found that the shear strength of CTC is largely stronger than the DTP’s. It is thanks to the addition of epoxy resin matrix, which increases the shear loading bearing to improve the strength of the final composites. The same ply stacking sequence and the injection of resin may have contributed to the similar stiffness of all CT’C, CTC samples, and non tufted composite samples. A large difference in the interlaminar stiffness property between the DTP and the CT’C/CTC is observed with the rigid samples. This can also be attributed to the presence of an epoxy resin matrix.

## 5. Conclusions

In the present paper, the interlaminar shear performance of multiscale was studied. This was due to the presence of the through the thickness tufting thread, reducing the shearing sliding of the laminated sample. Thereby, the interlaminar shear performance under mode II loading was improved. Several significant conclusions are listed as follows:

- A modified T steel shearing test was designed that can effectively study the pure interlaminar shear performance of tufted laminates under three tufting scales (DTP, CT’C, and CTC) in mode II condition;
- There was a positive proportional relationship of tufting density and interlaminar shear strength in DTP samples;
- CT’C samples presented an identical result with the non tufted composites, regardless of the various tufting densities;
- For CTC samples, with the tufting density increasing, only the shear strength increased. Meanwhile, the different tufting densities brought out both positive and negative effects on the interlaminar shear strength compared to the non tufted composites. The introduction of Z direction thread could degrade the interlaminar shear properties if the tufting density did not meet the need. The critical tufting spacing was 6 mm in the present study. Compared with DTP samples, the shear stiffness was improved with the infused resin, as was the shear strength and shear angle.

The present work focuses on the interlaminar shear strength under Mode II sliding loading. Therefore, fracture toughness can be subjected to testing, and the formula that links the tufting density and shear strength can be derived to predict the shear strength in future work. In addition, the analysis of the shear behavior of laminate composite in terms of energy accumulation and release is also interesting and can be applied to verify the present results.

## Figures and Tables

**Table 1:** Main properties of tufting threads.

Reference	Linear Density (tex)	Number of Filaments	Twist (T/m)
Tenax^®^ J HTA 40	2 × 67	2 × 1000	240 ± 16

**Table 2:** Description of test tufted samples.

Ref. of Samples	Tufting Spacing (mm)	Methods of Manufacture	Infused Resin System
DTP-T3	3	2-step tufting	—
DTP-T6	6		
DTP T9	9		
Non-tufted	—	—	LRI SICOMIN^®^ SR8200
CT’C T3	3	1-step tufting	
CT’C T6	6		
CT’C T9	9		
CTC T3	3	2-step tufting	
CTC T6	6		
CTC T9	9		

**Table 3:** Conclusive interlaminar shearing resistance results of all samples.

Ref. of Samples	Tufting Spacing (mm)	Shear Strength (MPa)	Shear Angle (rad)
DTP-T3	3	0.15 ± 0.03	1.17 ± 0.02
DTP-T6	6	0.10 ± 0.02	1.13 ± 0.02
DTP T9	9	0.05 ± 0.01	1.04 ± 0.01
Non-tufted	—	18.2 ± 0.18	0.28 ± 0.01
CT’C T3	3 (tufted without thread)	18.7 ± 0.23	0.28 ± 0.01
CT’C T6	6 (tufted without thread)	18.6 ± 0.23	0.28 ± 0.01
CT’C T9	9 (tufted without thread)	18.5 ± 0.32	0.28 ± 0.01
CTC T3	3	20.4 ± 0.81	0.30 ± 0.01
CTC T6	6	18.7 ± 0.58	0.27 ± 0.01
CTC T9	9	18.1 ± 0.23	0.26 ± 0.01

## Data Availability

Not applicable.
